# Effects of pH Value of the Electrolyte and Glycine Additive on Formation and Properties of Electrodeposited Zn-Fe Coatings

**DOI:** 10.1155/2013/273953

**Published:** 2013-06-11

**Authors:** İsmail Hakki Karahan

**Affiliations:** Department of Physics, Faculty of Science and Arts, Mustafa Kemal University, 31000 Hatay, Turkey

## Abstract

Environmentally friendly and cyanide-free sulfate bath under continuous current and the corrosion behavior of electrodeposits of zinc-iron alloys were studied by means of electrochemical tests in a solution of 3.5% NaCl in presence and absence of glycine. The effects of pH on the quality of Zn-Fe coatings were investigated in order to improve uniformity and corrosion protection performance of the coating films. The deposit morphology was analyzed using scanning electron microscopy (SEM), and X-ray diffraction (XRD) was used to determine the preferred crystallographic orientations of the deposits. It was found that the uniformity and corrosion resistance of Zn-Fe coating films were strongly associated with pH of the coating electrolyte. To obtain the effect of pH on the film quality and corrosion performances of the films, the corrosion test was performed with potentiodynamic anodic polarization method. It was also observed that uniformity and corrosion resistivity of the coating films were decreased towards pH = 5 and then improved with increasing pH value of the electrolyte. The presence of glycine in the plating bath decreases the corrosion resistance of Zn-Fe coatings.

## 1. Introduction

The electrodeposited Zn-Fe coatings possess many superior properties, such as excellent paintability, good welding properties, and good corrosion resistance, and have been considered to be an alternative for pure zinc coating of iron and steel products. However, there has been much interest in the use of electrodeposited zinc alloys for similar purposes. Zn-Fe coatings have attracted considerable attention in the automotive industry because they combine high corrosion resistance with excellent mechanical performance and improved capability for subsequent organic coatings [1, 2]. In addition, Zn-Fe alloy coatings with high iron content can serve as an effective undercoat for paints [3–7].

The Zn-Fe system is a very interesting one because besides the inducing behavior of Zn it presents the anomalous behavior of Fe. The anomalous codeposition model predicts that the less noble metal deposits preferentially. Anomalous codeposition is therefore a very important phenomenon in the electrodeposition of zinc alloys.

The intermediate of the less noble metal occupies more active surface sites of the substrate and inhibits the deposition of the other metal. In the aforementioned studies the coupled effects of the Zn-Fe alloy electrode position werenot described when both anomalous and induced systems occur together. 

The iron containing alloy results in a good adhesion to the substrate and allows application of these materials at higher temperatures. Therefore, many research groups have reported the preparation of these alloys with high or low iron content, respectively [8–16]. Recently, several research groups have reported on electrodeposition and characterization of Zn-Fe alloys; however, only a few works are focused on the effect of electrolyte pH on the structure and corrosion properties of Zn-Fe alloys. Depending on the electrolyte composition, temperature, current density, and pH of the solution, different properties can be obtained. This fact makes Zn-Fe alloys important to explore the effect of pH on the structure of these alloys. The effect of pH also changes the concentration of elements in the samples and distribution of outer shell electrons [17].

On the other hand, the use of additives in the electrodeposition bath is very vital due to their influence on the gain of an appropriate deposition potential and, growth and structure of the deposits obtained. The efficiency of the electrodeposition process and the characteristics of the Zn-Fe deposit can be improved by employing additives such as glycine in the plating bath. Generally, additives are added to bath at very little concentrations; their presence in the electrolyte promotes the formation of smooth and shiny coatings. The specific activity of an additive is generally understood in terms of its absorption onto the cathode surface during electrodeposition. Additive molecules adsorbed on the cathode surface can affect the activation energy [18] and the rate of charge transfer in the electrochemical reaction and may also influence the mechanism of electrocrystallization [19, 20]. In another previous study investigating the relation of corrosion properties and gelatin as additive in Zn-Fe alloys [21], the results indicated strong evidence of an increase in corrosion potential with adding gelatin in the electrolyte. An important part of metal electrodeposition processes is carried out from baths containing complexing agents. Recently, various complexing agents such as citrates, tartrates, fluoborates, sulfamates, gluconates, and glycinates have been used. These agents are nontoxic, easily obtained and, upon degradation, effluent treatment is easier.

The glycine has been used as a complexing agent in the electrodeposition of Zn-Ni [22], Cu-Co [23], Zn-Co [24, 25], Zn-Co-Cu [26], and Co-Ni-Mo alloys [27] and to obtain Zn [28]. These studies show that the deposits obtained from alkaline bath containing glycine are of high quality.

As glycine is not commonly used as additive to iron group or Zn alloys, a more detailed study is needed. Here, we report on structural and corrosion properties of electrodeposited Zn-Fe films investigated as a function of electrolyte pH and glycine as additive to provide more insight to the Zn-Fe alloying system and to better understand how glycine acts as an additive to electrodeposition solutions. 

## 2. Experimental Details

### 2.1. Electrodeposition of Zn-Fe Alloys

Zn_1−*x*_Fe_*x*_ alloys were prepared by electrodeposition under continuous current on aluminum and AISI 4140 steel disk substrates from a sulfate plating bath. The chemical composition of the AISI 4140 steel is 0.36C-0.80Mn-0.005Si-0.914Cr-0.30Ni-0.85Mo-0.075V-0.07S-0.143Cu-0.034P (wt%). The area of the substrates was (1.5 cm × 1.5 cm). Before the deposition, the substrates are prepared in the standard industrial way: chemical then electrolytic degreasing in sodium hydroxide solution (40 gL^−1^; 64.5°C), for 2 min, followed by water wash, mechanically grinding with silicon carbide papers from 3 to 0.5 Am and velvet, chemical pickling, and activation in an acid medium (hydrochloric acid; 30% in vol.) for 10 s, rinsed with the twice distilled water and then dried in air. So the wettability and therefore the reactivity of the substrate surface are increased. After these preparation steps, it is necessary to operate quickly to realize the electrodeposition, because the substrate surface may be spontaneously oxidized. Zn_1−*x*_Fe_*x*_ alloys were deposited from a sulfate plating bath which consisted of 40 g dm^−3^ ZnSO_4_·7H_2_O, 20 g dm^−3^ FeSO_4_·7H_2_O, 25 g dm^−3^ Na_3_C_6_H_5_O_7_, and 16 g dm^−3^ H_3_BO_3_. The pH value of the bath was varied in the range 3 to 6 using hydrochloric acid and NaOH. The employed electrolyte was prepared using p.a. chemicals (Merck) and double distilled water. Depositions were carried out at a current density of 1 A dm^−3^. Experimental temperature was 50°C. Counter electrode was a platinum electrode. The reference electrode used in all experiments was a saturated calomel electrode (SCE). 

The quantitative composition analysis of the electrodeposits at the surface was examined by using JEOL 6400 scanning electron microscope (SEM) with energy dispersive spectrometer (EDS) working at 15–30 kV. The preferred orientations of the deposits were determined by X-ray diffraction (XRD) analysis, using a Philips PANalytical X'Pert Pro X-ray diffractometer with CuK-*α* radiation (lambda = 1.5418 A). The 2*θ* range of 30–60° was recorded at a rate of 0.02° 2*θ*/0.5 s. The crystal phases were identified comparing the 2*θ* values and intensities. 

### 2.2. Corrosion Measurements

The electrochemical behaviors of the electrodeposited Zn-Fe alloys were analyzed in 3 wt% NaCl aqueous solution at room temperature in a Pyrex glass cell. The corrosion behaviors of the samples were investigated by a potentiodynamic polarization technique. Polarization measurements were performed with an electrochemical analyzer/workstation (Model 1100, CH Instruments, USA) with an anode, a cathode, and a reference electrode configuration. The exposed areas of the specimens were about 1 cm^2^. The specimens were covered with a cold setting resin and immersed into the solution until a steady open circuit potential (ocp) was reached. After equilibration, polarization was started at a rate of 1 mV/s.

## 3. Results and Discussion

To define the effect of electrolyte pH on the Zn-Fe alloy deposition process, cyclic voltammetry technique was used. 

**Figure 1:**
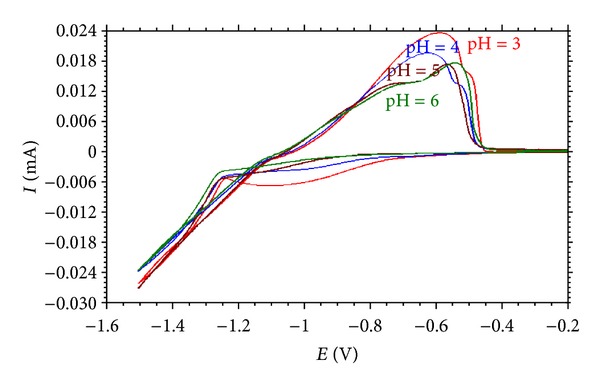
Cyclic voltammograms for different pH values.

Figure 1 shows the influence of pH values (3–6) of the plating electrolytes containing zinc, iron, and citrate ions on the potentiodynamic cathodic and anodic polarization parts of cyclic voltammogram curves. The potential was scanned from −0.2 V to the cathodic direction up to −1.5 V and ended at −0.2 V. The scan rate was 10 mVs^−1^ in all cases.

It is apparent from Figure 1 that the anodic part in the cyclic voltammograms consisted of one anodic current peak for the pH 3 and 4 and two anodic peaks for 5 and 6. This shows that the alloy dissolution takes place under two different potential values. In solutions, during the forward scan towards the negative direction, the cathodic current increased sharply when the deposition begins. The voltammograms suggest that codeposition of zinc and iron occurs at around −1.2 V. The limiting current peaks are more obvious in the cases of pH values 4, 5, and 6. A gradual shift of onset potential for the reduction of ions towards more negative direction is also observed. The shifting of onset potential for reduction at various pH values is influenced by protons in the case of low pH values and by hydroxide ions in the case of higher pH values. It was observed that in presence of more iron ions the cathodic current was increased but the cathodic peak potential was shifted less negatively in forward scan. So it was confirmed that in presence of more iron ions, the overall cathodic potential was reduced, due to the lowering of deposition rate by kinetic control processes. But the anodic peak potential was shifted less negatively. 

All the voltammograms show a gradual increase in the anodic current which results in the formation of “humps” on the anodic peaks. These “humps” are considered as the results of the dissolution of Fe and Zn, which forms on the surface of the deposit during cathodic scan. 

**Figure 2:**
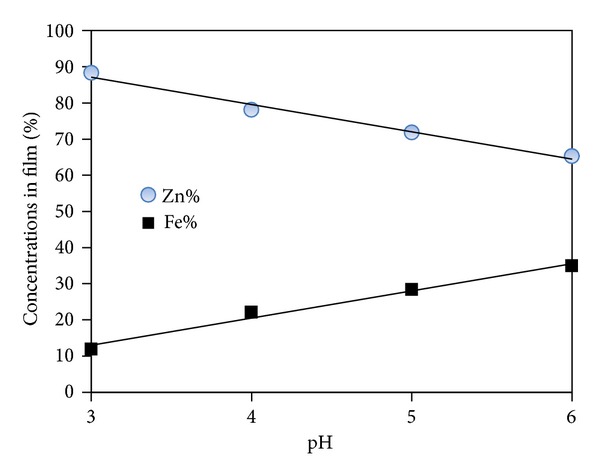
Content of the Zn and Fe in Zn-Fe alloy films.

Figure 1 also shows that the increase of the electrolyte pH caused a decrease of the dissolution peak. This peak corresponds to the preferential dissolution of zinc, so the decrease of dissolution peaks can be related to the composition of the dissolved deposit. It can be considered that an increase in the bath pH causes a decrease in the rate of zinc deposition, causing the observed decrease in size of dissolution peaks. The heights of the first anodic peaks generally increased with decreasing pH. The peak height is indicative of the amount or concentration of the given phase in the deposited film. Therefore, it is clear from Figure 2 that, with increasing the pH value, the amount of zinc constituent decreases as indicated by the relative decreases in the heights of the anodic peaks in the cyclic voltammograms.

Brenner [29] classified the electrodeposition of Zn-Fe alloys as anomalous. Codeposition of Zn and Fe is, however, not always anomalous since at low current densities, it is possible to obtain normal deposition, where Fe deposits preferentially to Zn. Figure 2 shows the dependence of the Fe and Zn concentrations in the films on the pH value of the electrolyte. Fe concentration of the coatings increased with increasing bath pH between values 3 and 6. 

**Figure 3:**
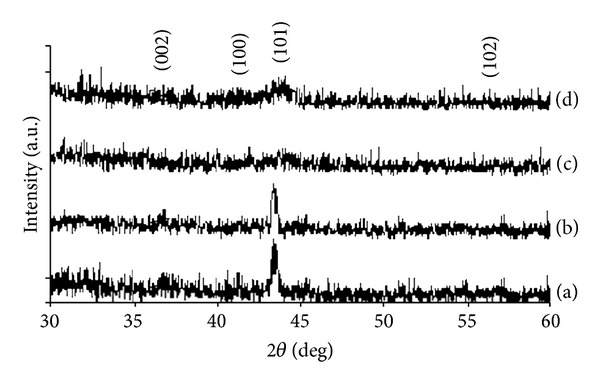
X-ray diffractograms of Zn-Fe alloys electrodeposited from sulfate bath. Deposition current density = 1 A·dm^−2^, *T* = 50°C. Bath pH: (a) pH = 3; (b) pH = 4; (c) pH = 5; (d) pH = 6.

The formation of Zn-Fe alloys was confirmed by XRD measurements.  Figure 3 shows the XRD patterns of zinc iron alloys prepared at different pH (pH 3–12). Each phase was identified by its characteristic diffraction peaks using JCPDS. The XRD analysis showed that the films are polycrystalline structure. The films deposited at different pH values exhibit a preferred orientation along the (002), (100), (101), and (102) planes. Generally, (101) hcp peak was present almost in all deposits with significant intensity.

Textural intensity was relatively higher for the deposits that obtained pH of 3. Certain peaks representing a specific crystallographic hkl plane gradually shifted either to higher or lower 2*θ* angles with the change in iron content in the deposit. The (101) peak shifted to higher 2*θ* when the iron content in the deposit was increased. 

In Figure 3, with pH value of the electrolyte changing from 3 to 5, the intensity of (101) preferred orientation was decreased, which led to the recession of corrosion resistance of the alloys. We can understand from the figure that increasing the pH decreased the (101) peaks until pH = 5, then it increased depending on the Fe content. From the XRD measurements, it was seen that the signals belonging to the *η* phase decreased as the electrolyte pH increased. 

The agreement of lattice parameters with reported values confirms that films are hexagonal and polycrystalline. The grain size (*D*) was calculated using Scherer's formula [30]

(1) $$\begin{array}{c} D=\frac{0.9\lambda }{\beta \;\cos\theta }. \end{array}$$

The microstrain was obtained by using the relation [31]

(2) $$\begin{array}{c} \varepsilon =\frac{\beta \cdot \cos\theta }{4}, \end{array}$$

where *β* is the full width at half maximum, *λ* is the wavelength of X-ray used (1.5400 Å), and *θ* is the diffraction angle.  Table 1 shows that these structural parameters are crucially dependent on the pH of the electrolyte solution. An increase in solution pH results in an increase in grain size. Increasing the bath pH from 3 to 5 significantly decreased the microstrain of the film. More increase in the bath pH caused the increase of microstrain. It is similar to SEM results.

**Figure 4:**
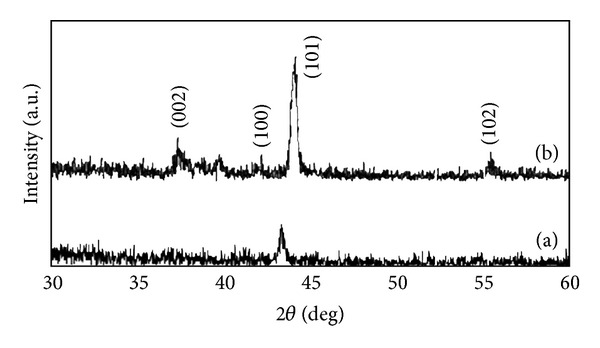
XRD spectra of Zn-Fe alloy coatings following deposition from different electrolytes: (a) without glycine, (b) containing glycine at pH = 3.

The X-ray patterns of Zn-Fe alloy coatings with and without glycine as additive are shown in Figures 4(a) and 4(b), respectively. The position of X-ray peaks shifted with adding glycine in the bath. For example, the (101) peaks were at 2*θ* = 42.87° for Zn-Fe coating without glycine and 2*θ* = 44.43° for Zn-Fe coating with glycine as additive. It is shown that addition of glycine generally provides a good rearrangement to the Zn-Fe alloys. 

**Figure 5:**
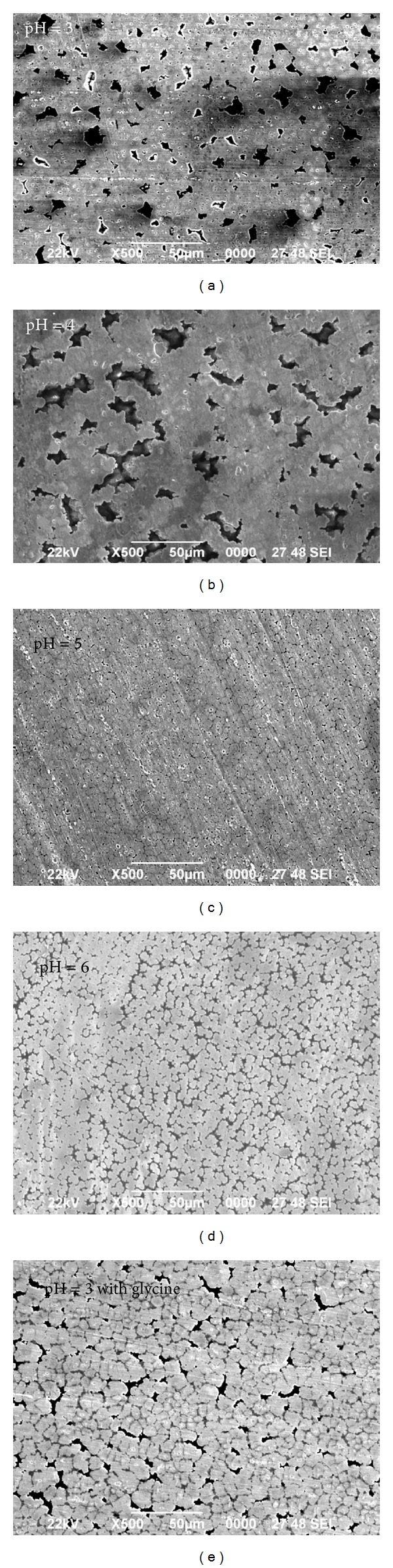
SEM images of the Zn-Fe films electrodeposited at different pH values of the electrolyte and pH = 3 Zn-Fe with glycine as additive.

After coating, samples were washed and dried with air for SEM observations.  Figure 5 shows the SEM images of the samples electrodeposited at different pH values of the electrolyte. The pH values were changed from 3 to 6 to investigate the effect of pH values on the deposits' properties. As can be seen in the figure, the average sizes of the grains have increased with increasing electrolyte pH values. Enlargement of film particles seems to lower the density of the coating films, that is, the number of grains per unit surface area. 

For the deposit obtained in the pH value of 5, compact deposit morphology was observed (Figure 5(c)). With further increases in the pH value, deposit with even larger grain size was observed (Figure 5(d)). It illustrates that the deposition at pH 5 and 6 had smooth and nonporous surfaces (Figures 5(c) and 5(d)). With decreasing pH values by < 5, the deposited films confirmed the formation of pore and cracked surface for Zn-Fe as shown in Figures 5(a) and 5(b). It can be concluded that a value of pH 5 and 6 is optimal for preparing bright, coherent, and uniform Zn-Fe binary alloy coatings. 

The increase of bath pH apparently modified the growth of iron nuclei, leading to the larger-grained deposits. It is understood from the figures that bath pH plays a major role in the grain-size refinement of Zn-Fe alloys. These results suggest that pH of the electrodeposition bath is closely associated with deposition properties such as average size of particles and density and compactness of resultant coating film. The Zn-Fe film obtained with glycine has finer grain size than the Zn-Fe alloy from glycine-free bath. The role of glycine as leveling agent for Zn-Fe codeposition is proposed. Finer grain size in Zn-Fe electrodeposited in the presence of glycine. The crystallographic orientations and grain size are both dependent on the presence of glycine.

**Figure 6:**
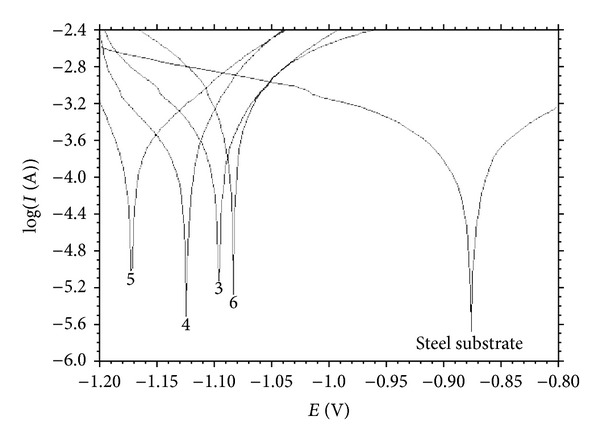
Potentiodynamic polarization measurements of electrodeposited Zn-Fe alloys in a 3.0 wt% NaCl aqueous solution.

Figure 6 shows the corrosion property of electrodeposited Zn-Fe alloy from sulfate bath in a 3 wt% NaCl aqueous solution. The corrosion potential Ecorr of the coating obtained at pH = 5 is the lowest corrosive, and the best corrosion resistance for Zn-Fe coatings was obtained in the pH value of 6. Compared with pH = 5 coating, it is found that the corrosion potential of the one deposited at pH = 6 alloy is 9% nobler. It is thus further concluded that the iron content of the Zn-Fe alloy coating up to pH value of 6 possesses superior anticorrosion behaviors than that of Zn-Fe coating having pH = 5. An increase of pH up to 5 increased the grain size of the Zn-Fe alloys, and, thereby, Zn-Fe alloys presented a lower degree of corrosion. 

Zinc is a good anticorrosive material, and the fundamental function of iron in the coatings is to make the corrosion potential more positive. In this case, the alloy coatings, become nobler than zinc coatings and the Zn-Fe alloy coatings become more corrosion resistant. Zn-Fe alloys have more negative corrosion potentials as the electrolyte pH, and so the grain size increases. 

**Figure 7:**
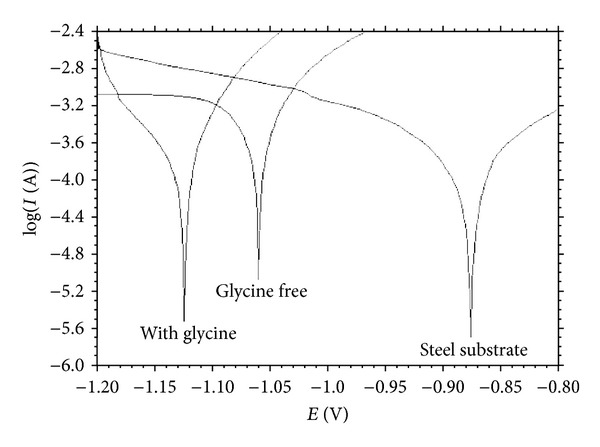
The effect of glycine on corrosion behavior of Zn-Fe electrodeposits in 3.0 wt% neutral NaCl solution.

Figure 7 shows the results corresponding to the influence of addition of 0.5 g L^−1^ glycine on the Zn-Fe alloy. The results show that the presence of glycine decreases corrosion resistance of the films. This behavior can be explained with the decrease of the microstrain in the film with adding glycine to the bath as an additive (Table 2).

## 4. Conclusions

In this study, we investigated various Zn-Fe alloys obtained from different pH values. The corresponding electroplating behavior and corrosion properties of Zn-Fe alloys were investigated using cyclic voltammetry and linear sweep voltammetry methods. The effects of bath pH and glycine addition to the electrolyte were investigated on the structure of Zn-Fe alloys and the corrosion behavior of AISI 4140 steel substrates. The average size of the grains increased with increase in pH of the electrolyte up to pH = 5, when microstrain decrease. pH of the coating bath was closely associated with uniformity and, thus, corrosion resistance of coating films. In this work, the best corrosion resistance for Zn-Fe coatings was obtained in the pH value of 6. The results show that the glycine as additive modifies the structure and surface topography of the deposits to a large extent and produces smoother deposits.

## Figures and Tables

**Table 1:** Structural parameters of Zn-Fe alloys electrodeposited at various pH values.

	pH value			
	3	4	5	6
Microstrain (*ε*) ×10^−2^	0.135	0.132	0.124	0.131
Grain size (nm)	100	102	130	128

**Table 2:** Structural parameters of Zn-Fe alloys electrodeposited at pH = 3 with and without glycine.

	Glycine free	With glycine
Microstrain (*ε*) ×10^−2^	0.135	0.179
Grain size (nm)	100	93
